# Supra­molecular inter­actions in a 1:1 co-crystal of acridine and 3-chloro­thio­phene-2-carb­oxy­lic acid

**DOI:** 10.1107/S2056989016005685

**Published:** 2016-04-08

**Authors:** Olakkandiyil Prajina, Packianathan Thomas Muthiah, Franc Perdih

**Affiliations:** a School of Chemistry, Bharathidasan University, Tiruchirappalli 620 024, Tamilnadu, India; b Faculty of Chemistry and Chemical Technology, University of Ljubljana, Vecna pot 113, PO Box 537, SI-1000 Ljubljana, Slovenia

**Keywords:** crystal structure, 3-chloro­thio­phene-2-carb­oxy­lic acid, acridine

## Abstract

In the title co-crystal, C_5_H_3_ClO_2_S·C_13_H_9_N, the components inter­act with each other *via* an O—H⋯N hydrogen bond. Acridine–acridine stacking, thio­phene–thio­phene stacking and acridine–thio­phene C—H⋯π inter­actions also occur in the crystal.

## Abstract 2

The asymmetric unit comprises one 3-chloro­thio­phene-2-carb­oxy­lic acid (3TPC) and one acridine mol­ecule linked together *via* an O—H⋯N hydrogen bond.

## Chemical context   

Co-crystals are solids in which two or more mol­ecules crystallize together and interact through non-covalent inter­actions (Odiase *et al.*, 2015 ▸). The study of non-covalent inter­actions in co-crystals not only adds to our knowledge but also has an undeniable relevance in the context of their pharmaceutical and biological inter­est (Chakraborty *et al.*, 2014 ▸; Desiraju, 1989 ▸). The main inter­actions concerned are various hydrogen bonding, π–π and C—H⋯π inter­actions (Aakeröy *et al.*, 2010 ▸). The acridine mol­ecule is a component present in anti­helminthic agents which are used in animals (Durchheimer *et al.*, 1980 ▸). Acridine derivatives also show *in vitro* activity against protozoa (Ngadi *et al.*, 1993 ▸). The acridine group is a well known inter­calator inter­acting with nucleobase pairs (Raju *et al.*, 2016 ▸; Nafisi *et al.*, 2007 ▸; Sazhnikov *et al.*, 2013 ▸). Acridine dyes are also widely used (Solovyeva *et al.*, 2014 ▸, Yasarawan *et al.*, 2011 ▸). Halogenated thio­phene carb­oxy­lic acid derivatives are the building blocks of many commercially available insecticides (Hull *et al.*, 2007 ▸). We extended our study on supra­molecular architectures in acridine mol­ecules with the investigation of the title co-crystal with 3-chloro­thio­phene-2-carb­oxy­lic acid (3TPC).
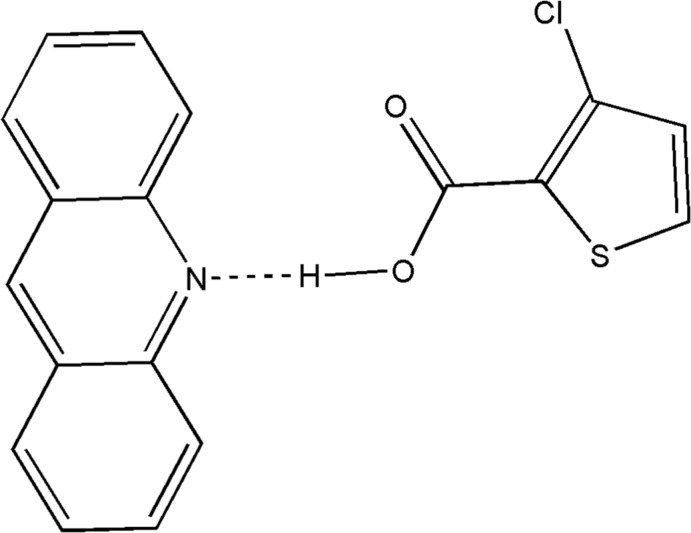


## Structural commentary   

The compound (**1**) is a 1:1 co-crystal of 3TPC and acridine. The inter­nal angle at N1 [C6—N1—C18 = 119.30 (15)°] and bond lengths [C18—N1 = 1.346 (2) and C6—N1 = 1.354 (2) Å] agree with those reported for neutral acridine structures (Aghabozorg *et al.*, 2011 ▸; Binder *et al.*, 1982 ▸; Goeta *et al.*, 2002 ▸). The two external bond angles at the carbon atom of the carboxyl group are 124.13 (17) and 110.75 (15)°. The high discrepancy between these two angles is typical of an unionized carboxyl group. The C=O distance of 1.316 (2) Å and C—OH distance of 1.199 (2) Å are also typical of the carboxyl group. These values also agree with the carb­oxy­lic acids reported in the literature (Kowalska *et al.*, 2015 ▸; Sienkiewicz-Gromiuk *et al.*, 2016 ▸). The dihedral angle between the carboxylic acid group and the thiophene ring is 9.01 (13)°. The bond distances and angles involving the thio­phene ring agree with those in structures reported earlier (Zhang *et al.*, 2014 ▸).

## Supra­molecular features   

**Figure 1:**
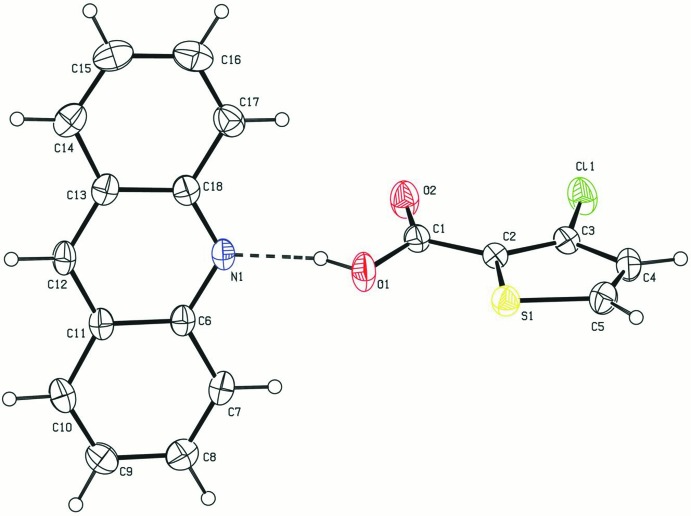
The asymmetric unit of the title compound, showing the atom-numbering scheme. Displacement ellipsoids are drawn at the 50% probability level. The dashed line represents the O—H⋯N hydrogen bond.

**Figure 2:**
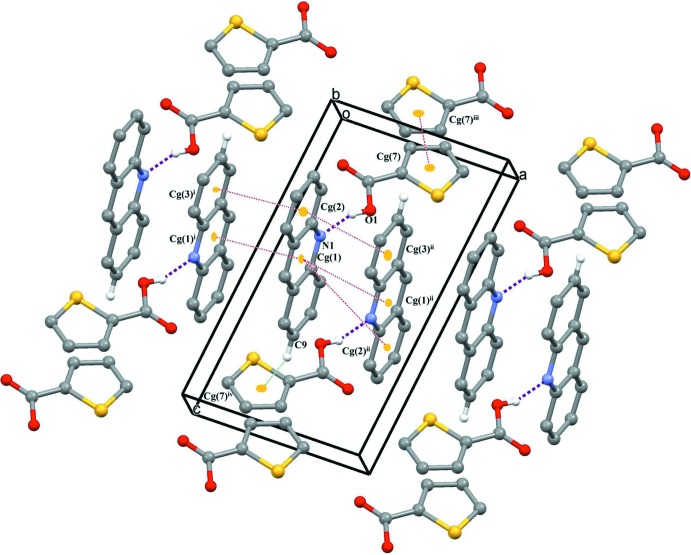
A view of the O—H⋯N hydrogen bonds (purple dashed lines), π–π stacking (acridine–acridine and thio­phene–thio­phene; red dashed lines) and C—H⋯π inter­actions between the acridine C—H group and the π-system of thio­phene (green dashed lines).

The 3TPC and acridine moieties are linked by an O—H⋯N hydrogen-bonding inter­action between (O1—H1) of the carboxyl group and the acridine nitro­gen atom (N1) (Table 1 ▸ and Fig. 1 ▸). This O—H⋯N hydrogen bond is reminiscent of the frequently used supra­molecular synthon in crystal engineering involving a carb­oxy­lic acid and a pyridine mol­ecule (Seaton, 2014 ▸; Lemmerer & Bernstein, 2010 ▸; Thomas *et al.*, 2010 ▸). A similar type of supra­molecular synthon is observed in a series of nine co-crystals involving acridine and benzoic acids (Kowalska *et al.*, 2015 ▸). This supra­molecular synthon is also present in the co-crystal of 5-chlorothiophene-2-carboxylic acid and acridine reported from our laboratory (Jennifer & Mu­thiah, 2014 ▸). This co-crystal and the title co-crystal differ only in the position of chlorine in the thio­phene ring. The hydrogen-bonded units are linked *via* π–π stacking inter­actions between the aromatic systems of acridine mol­ecules [*Cg*1⋯*Cg*1^i^ = 3.6419 (9), *Cg*1⋯*Cg*1^ii^ = 3.7526 (9), *Cg*1⋯*Cg*2^ii^ = 3.7293 (12), *Cg*2⋯*Cg*3^i^ = 3.6748 (12) and *Cg*2⋯*Cg*3^ii^ = 3.7298 (12) Å where *Cg*1 is the centroid of the N1/C6/C11/C12/C13/C18 ring, *Cg*2 is the centroid of the C6–C11 ring and *Cg*3 is the centroid of the C13–C18 ring; symmetry codes: (i) −*x*, 2 − *y*,1 − *z*; (ii) 1 − *x*, 2 − *y*,1 − *z*] and between the thio­phene rings [*Cg*7⋯*Cg*7^iii^ = 3.7611 (12) Å where *Cg*7 is the centroid of the thio­phene ring; symmetry code: (iii) 1 − *x*, 1 − *y*, −*z*]. The crystal structure also features C—H⋯π inter­actions, forming a three-dimensional supra­molecular architecture (Table 1 ▸ and Fig. 2 ▸).

## Database survey   

The crystal structures of a number of acridine co-crystals, acridinium salts and their metal complexes have been investigated in a variety of crystalline environments such as diphenic acid–acridine (1:1) (Shaameri *et al.*, 2001*a* ▸), 4,4′-bis­(hy­droxy­azo­benzene)–acridine (Chakraborty *et al.*, 2014 ▸), orcinol–acridine (1:2) and orcinol–acridine (1:1) co-crystal hydrate (Mukherjee *et al.*, 2011 ▸), acridinium isophthalate (Shaameri *et al.*, 2001*b* ▸) and acridinium 6-carb­oxy­pyridine-2- carboxyl­ate monohydrate (Derikvand *et al.*, 2011 ▸). A variety of metal complexes of acridine have also been reported (Ha, 2010 ▸, 2012 ▸; Sloufova & Slouf, 2000 ▸, 2001 ▸).

## Synthesis and crystallization   

To 10 ml of a hot methanol solution of 3TPC (40.6 mg, 25 mmol) were added 10 ml of a hot methano­lic solution of acridine (44.8 mg, 25 mmol). The resulting solution was warmed over a water bath for half an hour and then kept at room temperature for crystallization. After a week yellow plate-like crystals of (**1**) were obtained.

## Refinement   

Crystal data, data collection and structure refinement details are summarized in Table 2 ▸. All hydrogen atoms were readily located in difference Fourier maps and were subsequently treated as riding atoms in geometrically idealized positions, with C—H = 0.93 and O—H = 0.82 Å, and with *U*_iso_(H) = *kU*_eq_(C, O), where *k* = 1.5 for hy­droxy and 1.2 for all other H atoms.

## Supplementary Material

Crystal structure: contains datablock(s) I. DOI: 10.1107/S2056989016005685/hg5473sup1.cif

Structure factors: contains datablock(s) I. DOI: 10.1107/S2056989016005685/hg5473Isup2.hkl

Supporting information file. DOI: 10.1107/S2056989016005685/hg5473Isup3.cml

CCDC reference: 1472507

Additional supporting information:  crystallographic information; 3D view; checkCIF report

## Figures and Tables

**Table 1:** Hydrogen-bond geometry (Å, °) *Cg*7 is the centroid of the thio­phene ring.

*D*—H⋯*A*	*D*—H	H⋯*A*	*D*⋯*A*	*D*—H⋯*A*
O1—H1⋯N1	0.82	1.83	2.615 (2)	159
C9—H9⋯*Cg*7^i^	0.93	2.94	3.773 (2)	150

Symmetry code: (i) 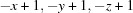.

**Table 2:** Experimental details

Crystal data	
Chemical formula	C_5_H_3_ClO_2_S·C_13_H_9_N
*M*_r_	341.80
Crystal system, space group	Triclinic, *P* 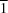
Temperature (K)	293
*a*, *b*, *c* (Å)	7.3371 (4), 8.3286 (5), 13.3819 (8)
α, β, γ (°)	107.577 (5), 97.706 (5), 93.953 (5)
*V* (Å^3^)	767.32 (8)
*Z*	2
Radiation type	Mo *K*α
μ (mm^−1^)	0.39
Crystal size (mm)	0.60 × 0.30 × 0.10

Data collection	
Diffractometer	Agilent SuperNova Dual Source diffractometer with an Atlas detector
Absorption correction	Multi-scan (*CrysAlis PRO*; Agilent, 2013 ▸)
*T*_min_, *T*_max_	0.813, 1.000
No. of measured, independent and observed [*I* > 2σ(*I*)] reflections	7182, 3516, 2722
*R*_int_	0.022
(sin θ/λ)_max_ (Å^−1^)	0.649

Refinement	
*R*[*F* ^2^ > 2σ(*F* ^2^)], *wR*(*F* ^2^), *S*	0.038, 0.109, 1.02
No. of reflections	3516
No. of parameters	209
H-atom treatment	H-atom parameters constrained
Δρ_max_, Δρ_min_ (e Å^−3^)	0.21, −0.23

Computer programs: *CrysAlis PRO* (Agilent, 2013 ▸), *SHELXS97* (Sheldrick, 2008 ▸), *SHELXL2014* (Sheldrick, 2015 ▸), *PLATON* (Spek, 2009 ▸) and *Mercury* (Macrae *et al.*, 2008 ▸), *PLATON* (Spek, 2009 ▸) and *publCIF* (Westrip, 2010 ▸).

## supplementary crystallographic information

### Crystal data

**Table:**

C_5_H_3_ClO_2_S·C_13_H_9_N	*Z* = 2
*M**_r_* = 341.80	*F*(000) = 352
Triclinic, *P*1	*D*_x_ = 1.479 Mg m^−^^3^
*a* = 7.3371 (4) Å	Mo *K*α radiation, λ = 0.71073 Å
*b* = 8.3286 (5) Å	Cell parameters from 2635 reflections
*c* = 13.3819 (8) Å	θ = 3.9–29.2°
α = 107.577 (5)°	µ = 0.39 mm^−^^1^
β = 97.706 (5)°	*T* = 293 K
γ = 93.953 (5)°	Plate, yellow
*V* = 767.32 (8) Å^3^	0.60 × 0.30 × 0.10 mm

### Data collection

**Table:**

Agilent SuperNova Dual Source diffractometer with an Atlas detector	3516 independent reflections
Radiation source: SuperNova (Mo) X-ray Source	2722 reflections with *I* > 2σ(*I*)
Detector resolution: 10.4933 pixels mm^-1^	*R*_int_ = 0.022
ω scans	θ_max_ = 27.5°, θ_min_ = 2.8°
Absorption correction: multi-scan (*CrysAlis PRO*; Agilent, 2013)	*h* = −9→8
*T*_min_ = 0.813, *T*_max_ = 1.000	*k* = −10→10
7182 measured reflections	*l* = −17→17

### Refinement

**Table:**

Refinement on *F*^2^	0 restraints
Least-squares matrix: full	Hydrogen site location: inferred from neighbouring sites
*R*[*F*^2^ > 2σ(*F*^2^)] = 0.038	H-atom parameters constrained
*wR*(*F*^2^) = 0.109	*w* = 1/[σ^2^(*F*_o_^2^) + (0.0488*P*)^2^ + 0.133*P*] where *P* = (*F*_o_^2^ + 2*F*_c_^2^)/3
*S* = 1.02	(Δ/σ)_max_ < 0.001
3516 reflections	Δρ_max_ = 0.21 e Å^−^^3^
209 parameters	Δρ_min_ = −0.23 e Å^−^^3^

### Special details

**Table:**

Geometry. All esds (except the esd in the dihedral angle between two l.s. planes) are estimated using the full covariance matrix. The cell esds are taken into account individually in the estimation of esds in distances, angles and torsion angles; correlations between esds in cell parameters are only used when they are defined by crystal symmetry. An approximate (isotropic) treatment of cell esds is used for estimating esds involving l.s. planes.

### Fractional atomic coordinates and isotropic or equivalent isotropic displacement parameters (Å^2^)

**Table:**

	*x*	*y*	*z*	*U*_iso_*/*U*_eq_	
Cl1	0.21228 (7)	0.16757 (7)	−0.02749 (4)	0.05748 (17)	
S1	0.69521 (6)	0.48402 (6)	0.14957 (4)	0.04516 (15)	
O1	0.42177 (19)	0.63242 (19)	0.26579 (11)	0.0581 (4)	
H1	0.3486	0.6911	0.2968	0.087*	
O2	0.16569 (18)	0.49072 (19)	0.15614 (11)	0.0569 (4)	
N1	0.26273 (19)	0.85354 (18)	0.39993 (11)	0.0390 (3)	
C1	0.3309 (2)	0.5158 (2)	0.18026 (14)	0.0392 (4)	
C2	0.4606 (2)	0.4179 (2)	0.11735 (13)	0.0366 (4)	
C3	0.4277 (2)	0.2754 (2)	0.03048 (14)	0.0403 (4)	
C4	0.5886 (3)	0.2201 (3)	−0.00919 (16)	0.0499 (5)	
H4	0.5890	0.1248	−0.0675	0.060*	
C5	0.7426 (3)	0.3226 (3)	0.04802 (16)	0.0512 (5)	
H5	0.8614	0.3061	0.0331	0.061*	
C6	0.2995 (2)	0.8560 (2)	0.50232 (14)	0.0367 (4)	
C7	0.3599 (2)	0.7110 (2)	0.52526 (16)	0.0451 (4)	
H7	0.3756	0.6162	0.4701	0.054*	
C8	0.3948 (3)	0.7096 (3)	0.62689 (17)	0.0508 (5)	
H8	0.4331	0.6131	0.6407	0.061*	
C9	0.3742 (3)	0.8518 (3)	0.71176 (16)	0.0505 (5)	
H9	0.3994	0.8485	0.7811	0.061*	
C10	0.3179 (3)	0.9933 (3)	0.69378 (15)	0.0470 (5)	
H10	0.3050	1.0864	0.7508	0.056*	
C11	0.2784 (2)	1.0008 (2)	0.58805 (14)	0.0374 (4)	
C12	0.2206 (2)	1.1415 (2)	0.56483 (14)	0.0406 (4)	
H12	0.2070	1.2375	0.6197	0.049*	
C13	0.1826 (2)	1.1413 (2)	0.45997 (15)	0.0397 (4)	
C14	0.1250 (3)	1.2834 (3)	0.43165 (18)	0.0521 (5)	
H14	0.1088	1.3814	0.4843	0.063*	
C15	0.0936 (3)	1.2765 (3)	0.3283 (2)	0.0606 (6)	
H15	0.0577	1.3706	0.3104	0.073*	
C16	0.1148 (3)	1.1285 (3)	0.24777 (19)	0.0631 (6)	
H16	0.0921	1.1263	0.1773	0.076*	
C17	0.1677 (3)	0.9888 (3)	0.27050 (16)	0.0528 (5)	
H17	0.1788	0.8915	0.2159	0.063*	
C18	0.2061 (2)	0.9916 (2)	0.37835 (14)	0.0398 (4)	

### Atomic displacement parameters (Å^2^)

**Table:**

	*U*^11^	*U*^22^	*U*^33^	*U*^12^	*U*^13^	*U*^23^
Cl1	0.0514 (3)	0.0565 (3)	0.0477 (3)	−0.0036 (2)	0.0025 (2)	−0.0040 (2)
S1	0.0407 (3)	0.0458 (3)	0.0437 (3)	0.00472 (19)	0.00429 (19)	0.0075 (2)
O1	0.0474 (8)	0.0552 (9)	0.0509 (8)	0.0084 (7)	0.0069 (6)	−0.0141 (7)
O2	0.0397 (8)	0.0638 (10)	0.0539 (9)	0.0063 (6)	0.0081 (6)	−0.0012 (7)
N1	0.0361 (8)	0.0383 (8)	0.0363 (8)	0.0043 (6)	0.0094 (6)	0.0008 (7)
C1	0.0435 (10)	0.0365 (10)	0.0355 (9)	0.0045 (7)	0.0057 (7)	0.0085 (8)
C2	0.0401 (9)	0.0374 (10)	0.0318 (9)	0.0077 (7)	0.0050 (7)	0.0096 (8)
C3	0.0442 (10)	0.0398 (10)	0.0339 (9)	0.0052 (7)	0.0035 (7)	0.0082 (8)
C4	0.0549 (12)	0.0478 (12)	0.0415 (10)	0.0136 (9)	0.0119 (9)	0.0027 (9)
C5	0.0465 (11)	0.0576 (13)	0.0505 (12)	0.0165 (9)	0.0154 (9)	0.0132 (10)
C6	0.0282 (8)	0.0371 (10)	0.0405 (10)	0.0006 (6)	0.0098 (7)	0.0049 (8)
C7	0.0423 (10)	0.0379 (10)	0.0516 (11)	0.0065 (8)	0.0148 (8)	0.0057 (9)
C8	0.0464 (11)	0.0514 (12)	0.0594 (13)	0.0073 (9)	0.0118 (9)	0.0227 (11)
C9	0.0502 (11)	0.0574 (13)	0.0436 (11)	−0.0018 (9)	0.0069 (8)	0.0175 (10)
C10	0.0491 (11)	0.0457 (11)	0.0376 (10)	−0.0037 (8)	0.0092 (8)	0.0015 (9)
C11	0.0303 (8)	0.0360 (9)	0.0386 (9)	−0.0029 (7)	0.0078 (7)	0.0016 (8)
C12	0.0339 (9)	0.0341 (10)	0.0432 (10)	−0.0011 (7)	0.0092 (7)	−0.0038 (8)
C13	0.0273 (8)	0.0377 (10)	0.0497 (11)	−0.0001 (7)	0.0074 (7)	0.0074 (8)
C14	0.0380 (10)	0.0440 (12)	0.0718 (14)	0.0030 (8)	0.0063 (9)	0.0158 (11)
C15	0.0440 (11)	0.0639 (15)	0.0809 (17)	0.0051 (10)	0.0024 (11)	0.0366 (14)
C16	0.0498 (12)	0.0863 (18)	0.0580 (14)	0.0021 (11)	0.0013 (10)	0.0343 (14)
C17	0.0444 (11)	0.0654 (14)	0.0438 (11)	0.0043 (9)	0.0050 (8)	0.0116 (10)
C18	0.0278 (8)	0.0465 (11)	0.0405 (10)	0.0012 (7)	0.0059 (7)	0.0075 (8)

### Geometric parameters (Å, º)

**Table:**

Cl1—C3	1.7207 (18)	C8—H8	0.9300
S1—C5	1.692 (2)	C9—C10	1.352 (3)
S1—C2	1.7261 (17)	C9—H9	0.9300
O1—C1	1.316 (2)	C10—C11	1.427 (3)
O1—H1	0.8200	C10—H10	0.9300
O2—C1	1.199 (2)	C11—C12	1.379 (2)
N1—C18	1.346 (2)	C12—C13	1.393 (2)
N1—C6	1.354 (2)	C12—H12	0.9300
C1—C2	1.478 (3)	C13—C14	1.421 (3)
C2—C3	1.368 (3)	C13—C18	1.426 (3)
C3—C4	1.408 (3)	C14—C15	1.355 (3)
C4—C5	1.353 (3)	C14—H14	0.9300
C4—H4	0.9300	C15—C16	1.404 (3)
C5—H5	0.9300	C15—H15	0.9300
C6—C7	1.418 (2)	C16—C17	1.357 (3)
C6—C11	1.426 (2)	C16—H16	0.9300
C7—C8	1.354 (3)	C17—C18	1.426 (3)
C7—H7	0.9300	C17—H17	0.9300
C8—C9	1.405 (3)		
			
C5—S1—C2	92.22 (9)	C8—C9—H9	119.6
C1—O1—H1	109.5	C9—C10—C11	120.52 (19)
C18—N1—C6	119.31 (15)	C9—C10—H10	119.7
O2—C1—O1	125.12 (18)	C11—C10—H10	119.7
O2—C1—C2	124.13 (17)	C12—C11—C6	118.43 (16)
O1—C1—C2	110.75 (15)	C12—C11—C10	123.13 (17)
C3—C2—C1	130.54 (16)	C6—C11—C10	118.44 (17)
C3—C2—S1	109.54 (14)	C11—C12—C13	120.66 (17)
C1—C2—S1	119.91 (13)	C11—C12—H12	119.7
C2—C3—C4	113.99 (17)	C13—C12—H12	119.7
C2—C3—Cl1	124.70 (15)	C12—C13—C14	122.99 (18)
C4—C3—Cl1	121.31 (15)	C12—C13—C18	117.77 (16)
C5—C4—C3	111.64 (19)	C14—C13—C18	119.23 (17)
C5—C4—H4	124.2	C15—C14—C13	120.2 (2)
C3—C4—H4	124.2	C15—C14—H14	119.9
C4—C5—S1	112.61 (15)	C13—C14—H14	119.9
C4—C5—H5	123.7	C14—C15—C16	120.6 (2)
S1—C5—H5	123.7	C14—C15—H15	119.7
N1—C6—C7	119.41 (16)	C16—C15—H15	119.7
N1—C6—C11	121.65 (16)	C17—C16—C15	121.5 (2)
C7—C6—C11	118.94 (16)	C17—C16—H16	119.2
C8—C7—C6	120.47 (19)	C15—C16—H16	119.2
C8—C7—H7	119.8	C16—C17—C18	119.8 (2)
C6—C7—H7	119.8	C16—C17—H17	120.1
C7—C8—C9	120.91 (19)	C18—C17—H17	120.1
C7—C8—H8	119.5	N1—C18—C17	119.21 (18)
C9—C8—H8	119.5	N1—C18—C13	122.18 (16)
C10—C9—C8	120.73 (18)	C17—C18—C13	118.61 (17)
C10—C9—H9	119.6		
			
O2—C1—C2—C3	−7.7 (3)	C7—C6—C11—C12	179.62 (14)
O1—C1—C2—C3	171.99 (17)	N1—C6—C11—C10	179.36 (15)
O2—C1—C2—S1	170.91 (14)	C7—C6—C11—C10	−0.4 (2)
O1—C1—C2—S1	−9.4 (2)	C9—C10—C11—C12	179.99 (16)
C5—S1—C2—C3	0.03 (13)	C9—C10—C11—C6	0.0 (3)
C5—S1—C2—C1	−178.87 (14)	C6—C11—C12—C13	0.5 (2)
C1—C2—C3—C4	178.94 (16)	C10—C11—C12—C13	−179.48 (15)
S1—C2—C3—C4	0.2 (2)	C11—C12—C13—C14	−179.16 (15)
C1—C2—C3—Cl1	−1.6 (3)	C11—C12—C13—C18	−0.2 (2)
S1—C2—C3—Cl1	179.68 (10)	C12—C13—C14—C15	178.67 (17)
C2—C3—C4—C5	−0.4 (2)	C18—C13—C14—C15	−0.2 (3)
Cl1—C3—C4—C5	−179.89 (13)	C13—C14—C15—C16	0.9 (3)
C3—C4—C5—S1	0.4 (2)	C14—C15—C16—C17	−0.3 (3)
C2—S1—C5—C4	−0.25 (16)	C15—C16—C17—C18	−1.1 (3)
C18—N1—C6—C7	−179.75 (14)	C6—N1—C18—C17	179.93 (15)
C18—N1—C6—C11	0.5 (2)	C6—N1—C18—C13	−0.2 (2)
N1—C6—C7—C8	−179.02 (16)	C16—C17—C18—N1	−178.39 (17)
C11—C6—C7—C8	0.7 (2)	C16—C17—C18—C13	1.8 (3)
C6—C7—C8—C9	−0.7 (3)	C12—C13—C18—N1	0.1 (2)
C7—C8—C9—C10	0.3 (3)	C14—C13—C18—N1	179.06 (14)
C8—C9—C10—C11	0.0 (3)	C12—C13—C18—C17	179.95 (15)
N1—C6—C11—C12	−0.6 (2)	C14—C13—C18—C17	−1.1 (2)

### Hydrogen-bond geometry (Å, º)

*Cg*7 is the centroid of the thiophene ring.

**Table:**

*D*—H···*A*	*D*—H	H···*A*	*D*···*A*	*D*—H···*A*
O1—H1···N1	0.82	1.83	2.615 (2)	159
C9—H9···*Cg*7^i^	0.93	2.94	3.773 (2)	150

Symmetry code: (i) −*x*+1, −*y*+1, −*z*+1.
